# Selection and characterization of salt-tolerant plant growth promoting bacteria associated with the endosphere and rhizosphere of perennial glasswort from the Apulia Region (Italy)

**DOI:** 10.3389/fpls.2026.1718440

**Published:** 2026-03-05

**Authors:** Angela Guerrieri, Angela Racioppo, Beatriz García-Béjar, María Arévalo-Villena, Antonio Bevilacqua, Milena Sinigaglia, Barbara Speranza, Maria Rosaria Corbo

**Affiliations:** 1 Department of the Science of Agriculture, Food, Natural Resources and Engineering, University of Foggia, Foggia, Italy; 2 Department of Analytical Chemistry and Food Technology, University of Castilla-La Mancha, Ciudad Real, Spain

**Keywords:** endosphere, halophytes, marginal areas, plant growth promoting bacteria, rhizosphere

## Abstract

Soil salinization threatens global agricultural productivity, making halotolerant plant growth promoting bacteria (PGPB) crucial for sustainable farming in marginal areas. This study isolated and characterized PGPB from perennial glasswort (*Arthrocaulon macrostachyum*) collected at two sites in Margherita di Savoia, Apulia (Italy), during spring 2023. From rhizosphere and endosphere samples, 110 bacterial isolates (100 rhizobacteria, 10 endophytes) were obtained and characterized. Functional screening revealed: 18 isolates (16%) capable of phosphate solubilization, 25 isolates (23%) for silicon solubilization, 20 isolates (18%) producing indole acetic acid, 34 isolates (31%) producing siderophores, and 50 isolates (45%) demonstrating salt tolerance above 10% NaCl, with 28 isolates (25%) tolerating concentrations up to 17.5%. Using RAPD-PCR differentiation and Principal Component Analysis of PGPB traits, three promising halotolerant candidates were selected: *Pseudomonas* sp. (105S), *Bacillus safensis* (80S), and *Peribacillus frigotolerans* (114S), each exhibiting complementary plant growth promoting characteristics. These isolates represent valuable candidates for future field validation as biofertilizers in salt-affected agricultural systems.

## 1 Introduction

The growing global population presents significant challenges for agricultural sustainability, with food demand projected to increase by 70% by 2050 (Dardanelli et al., 2010). Meeting this demand requires substantial increases in crop production under increasingly constrained conditions. Current agricultural practices heavily rely on nitrogen-based fertilizers to boost yields; however, this approach has severe environmental consequences, including soil fertility degradation and ecosystem contamination (Barzallo et al., 2025).

Soil salinization compounds these challenges, as salt accumulation progressively alters soil composition, leading to widespread soil degradation and declining crop productivity (FAO, 2016; Mishra and Tanna, 2017). The problem is particularly serious given that over 20% of the world’s land is already affected by salinity, rendering vast areas unsuitable for conventional agriculture (Mukhtar et al., 2018; Szymańska et al., 2016). As arable land becomes increasingly scarce, the need to utilize marginal environments, such as salt-affected coastal zones, becomes more urgent. This reality requires innovative approaches to enhance crops’ resilience to abiotic stresses, particularly salinity, thereby unlocking the agricultural potential of previously unsuitable land.

The rhizosphere, the part of the soil surrounding the roots, and its two sub-compartments, the rhizosplane (root surface) and the endosphere (internal root tissue), are areas of plants with the greatest microbial diversity (Shah et al., 2021). These compartments house a variety of Plant Growth Promoting Bacteria (PGPB), which play a crucial role in solubilizing essential soil nutrients such as phosphorus (P), iron (Fe), and potassium (K). PGPBs also contribute to plant resistance against pathogens and diseases and enhance crop yields (Barzallo et al., 2025; Lee, 2023). Recent studies have demonstrated that some genera of these beneficial microbes (*Pseudomonas*, *Bacillus*, *Azospirillum*, and *Streptomyces*) could potentially replace chemical fertilizers by improving the carbon cycle and reducing carbon losses from soil (Wang et al., 2021; Racioppo et al., 2023; Alzate Zuluaga et al., 2024; Khosravi et al., 2024; Xie et al., 2024; Zahra et al., 2024; Guerrieri et al., 2025; Jiang et al., 2025; Li et al., 2025).

Among halophytic plants perennial glasswort (*Arthrocaulon macrostachyum*) is abundantly present in the coastal areas, where it can play a key role in the ecosystem stability, as it has developed strategies for growing in saline environments. This glasswort species is a Macaronesian Mediterranean distribution taxon that prefers salty coastal places with salinity higher than marine and often with a rocky substrate. It is in salt marshes, in sites not directly affected by tide variations, where it grows on the reliefs between the ponds (Cárdenas-Pérez et al., 2020, 2021; Duri et al., 2025; Loconsole et al., 2019). For instance, the Margherita di Savoia salt marshes, in north-eastern Apulia (Barletta-Andria-Trani province, Apulia, Italy), provide an ideal natural habitat for these plants where they grow spontaneously and thrive, making this area a rich and unexplored source of beneficial microbes resistant to high salt concentrations.

Recent studies on *Salicornia* have identified halotolerant PGPB (*Pseudomonas, Bacillus, Halomonas*) with phosphate solubilization and biocontrol activities (Álvarez-Hubert et al., 2024; Sánchez et al., 2025; Teles et al., 2024), demonstrating their biotechnological potential. Furthermore, recent studies on *Salicornia ramosissima* have demonstrated that biofertilization with marine PGPB significantly enhances drought tolerance, with inoculated plants showing reduced water loss (77% vs 59% in non-inoculated plants under extreme drought) and improved photochemical performance (Cruz de Carvalho et al., 2025). These findings collectively underscore the immense biotechnological potential of halophyte-associated PGPB for developing sustainable agricultural practices in marginal and salt-affected environments.

Despite the increasing interest in plant-microbiome interactions, microbial communities in halophyte plants growing in coastal environments remain poorly characterized. This knowledge is essential for understanding how these plants maintain beneficial microbial associations under fluctuating abiotic stress conditions, providing a first insight into their strategies for coping with salt stress, drought, and nutrient limitation-conditions that are relevant under climate change scenarios. Moreover, characterizing microbial dynamics in halophytes may reveal beneficial microorganisms with biotechnological potential for coastal agriculture, phytoremediation, and biosaline applications, thus bridging fundamental ecology and applied biotechnology in saline environments.

On this basis, the present study aimed to isolate, characterize, and select salt-tolerant potential PGPB from perennial glasswort *(A. macrostachyum)* collected at two sites located in the coastal dunes of Margherita di Savoia. Microbiological sampling was conducted in triplicate in 2023. After isolation, all the obtained strains were characterized using morphological, biochemical, and genetic approaches.

## 2 Materials and methods

### 2.1 Sample collection and preparation

Microbiological sampling of perennial glasswort *(A. macrostachyum)* was performed in triplicate during the spring season (March 2023). All samples were collected in two sites located in Margherita di Savoia (S1: N 41.25.39.51, E 16.0.28.46; S2: N 41.42.76.54, E 16.00.79.45), a coastal area that is characterized by high salinity and became a nature reserve in 1977 because it has a unique ecosystem and considerable biodiversity. Both sites were located in theBarletta-Andria-Trani area in the north-eastern Apulia (Italy), within coastal marsh wetlands, characterized by soils ranging from predominantly sandy to clayey, with rare rocky areas (Duri et al., 2025). Each sample was collected as described by Yamamoto et al. (2018). Namely, for each plant, two types of samples were collected: first, a 20x20 cm area at a depth of 10–20 cm was demarcated to collect the surrounding soil; then, the plant was gently uprooted, including the entire root system. All collected material was stored in a portable refrigerator at 4°C, transported immediately to the laboratory, and refrigerated until further analysis. On the day of analysis, three distinct sample types were recovered from each plant to target different microbial compartments: bulk soil, rhizosphere-associated root segments, and internal plant tissues (endosphere). Sample processing for each compartment is detailed in the following sections.

### 2.2 Metagenomic profiling of soil

Soil samples (20 g) were collected in triplicate from each sampling site and subjected to shotgun metagenomic sequencing using the Illumina short-read NovaSeq platform at Aurogene s.r.l. (Rome, Italy). DNA extraction was performed using the DNeasy PowerSoil Pro Kit (Qiagen, Hilden, Germany) according to the manufacturer’s instructions. Library preparation was conducted using the Nextera DNA Flex Library Prep Kit (Illumina, San Diego, CA, USA), and sequencing was performed with 2×150 bp paired-end reads, targeting a sequencing depth of approximately 10 Gb per sample (equivalent to ~66 million paired-end reads). Raw sequencing data (fastq files) were preprocessed using the fastP tool (v0.20.1) to remove adapter-contaminated sequences and low-quality reads (below average quality 30, minimum length 100 bp). Quality control metrics were assessed before and after filtering to ensure data integrity.

Downstream bioinformatic analyses were performed on the high-quality clean data. Filtered reads were processed with Kraken2 (v2.1.2) for taxonomic classification, followed by Bracken (v2.9) for species abundance quantification in the DNA sequences. Bracken utilized the PlusPFP database (containing RefSeq archaea, bacteria, viral, plasmid, human1, UniVec Core, protozoa, fungi, and plant sequences) available at https://benlangmead.github.io/aws-indexes/k2 to estimate the number of reads originating from each species detected in the individual metagenomic samples. Taxonomic abundances obtained from Bracken outputs were visualized using the Krona tool (v2.8.1).

### 2.3 Isolation of endophytic microorganisms

Endophytic cell isolation was carried out following Christakis et al. (2021) with some modifications. The sample type comprised the apical portion of the plant, including leaves and a small section (approximately 1 cm) of root tissue. Plant material was first repeatedly washed with sterile distilled water to remove soil and dust particles. Surface sterilization was then performed through sequential treatments: 75% (v/v) ethanol for 60 s, 3% (w/v) sodium hypochlorite for 10 min, 75% (v/v) ethanol for 60 s, followed by ten rinses with sterile distilled water (dH_2_O). To confirm sterilization efficiency, 100 μL of the final rinse water from each sample was plated on Nutrient Agar (NA) and incubated at 28°C for 48 h; all control plates showed no microbial growth, confirming successful surface sterilization. Only plant material passing this sterility check was used further. Successfully sterilized tissues (approximately 500 mg per sample) were aseptically sliced with a sterile scalpel and ground into a slurry using an autoclaved pestle and mortar. The slurry was transferred into sterile bottles containing 30 mL of autoclaved dH_2_O and incubated on a rotary shaker (150 rpm, 25°C, 2 h) to release endophytic cells. Subsequently, 100 μl aliquots were plated in triplicate on NA and incubated at 28°C for 48h.

Morphologically distinct colonies were selected, purified, grown in Nutrient Broth (NB), and stored on NA slants at 4°C.

### 2.4 Isolation of rhizosphere microorganisms

The sample type consisted of 3 cm root portions with tightly adhering soil particles, representing the rhizosphere compartment. After gently shaking the roots to detach adhering particles loosely, soil adhering tightly to the root surface (0–3 mm zone) was carefully collected with sterile spatulas. Each sample (10 g) was then homogenized for 1 min in 90 mL of sterile 0.9% NaCl solution by a Stomacher Lab Blender 400 (Seward, London, England). Then, serial dilutions were carried out and plated onto appropriate medium, to select and count mesophilic bacteria (Nutrient agar; 30°C for 48 h), *Pseudomonas* spp. (Pseudomonas Agar Base added with Pseudomonas Selective Supplement; 25°C for 48–72 h), spore-formers bacteria (PCA, after heat-treating the dilutions at 80°C for 10 min; the plates were incubated at 30°C for 24 h), Actinobacteria (Bacteriological Peptone, 10 g/L; Beef Extract, 5 g/L; NaCl, 5 g/L; Glycerol, 10 g/L; Agar, 20 g/L; 22°C for 7 days), and nitrogen-fixing bacteria (Glucose, 5 g/L; K_2_HPO_4_, 0.8 g/L; MgSO_4_, 0.2 g/L; FeSO_4_, 0.04 g/L; Na_2_MoO_4_, 0.005 g/L; CaCl_2_ Anhydrous, 0.15 g/L; Agar, 15 g/L; 30°C for 5 days; 30°C for 5 days). All media and supplements were from Oxoid (Milan, Italy). From each plate, 5 to 10 colonies with different morphologies were randomly selected, isolated, purified, labeled with a numeric code, and stored at 4°C.

### 2.5 Characterization tests

All isolates (both rhizobacteria and endophytes) were morphologically and biochemically characterized through Gram staining, catalase, oxidase, urease test, microscopic observation, spore production, and motility (Olsen and Sommers, 1982).

To assess the presence of plant growth promoting traits, the isolates were tested for their capacity to solubilize phosphate and silicon, produce indole acetic acid and siderophores, generate ammonia, resist drought and high salinity.

The ability of microbial isolates to produce ammonium was evaluated using the method described by Kumar et al. (2015), with some modifications. A 100 µL bacterial preculture was inoculated into test tubes containing Peptone Water medium (10.0 g peptone, 5.0 g NaCl, 1000 mL distilled water, pH 7.0). Tubes were incubated for 96 h at 30°C for mesophilic and spore-forming bacteria, 22°C for Actinobacteria, and 25°C for *Pseudomonas* spp. Ammonium production was determined by adding 0.5 mL of Nessler’s reagent to each tube. A pale-yellow color indicated low ammonium production, while a yellow-brown or orange color indicated high ammonium production.

Phosphate-solubilizing bacteria were assessed on Pikovsky medium (10 g/L d-glucose, 5 g/L Ca_3_(PO_4_)_2_, 0.5 g/L (NH_4_)_2_SO_4_, 0.2 g/L KCl, 20 g/L agar, 1000 mL distilled water, pH 7.0, Oxoid) following Dawwam et al. (2013). Plates were inoculated and incubated at 30°C for 48 hours. Phosphate solubilization was indicated by a clear zone around the inoculum.

Siderophore production was evaluated using the method of Battini et al. (2016), with some modifications. Each microbial isolate was spot-inoculated (20 µL) on TSA (Tryptone Soy Agar, Oxoid) plates and incubated at 28°C for 48 hours. After incubation, 10 mL of CAS (Chromeazurol S) blue agar was overlaid on the plates incubated at 28°C for 7 days. Siderophore-producing strains were identified by a color change from blue to yellow or orange around the colonies.

The silicon solubilization test was performed using a specific medium containing 10 g/L of glucose, 2.5 g/L Mg_2_O_8_Si_3_, 20 g/L agar, and 1 L of distilled water. Bacterial strains were spot-inoculated (20 µL) in the medium and incubated for 7 days at 28°C (Kang et al., 2017). After incubation, the presence of a transparent halo indicates the strain’s ability to solubilize silicon (Raturi et al., 2021).

Indole acetic acid (IAA) production was assessed using Yeast Extract Mannitol medium (YEM, 1 g/L Yeast Extract, 10 g/L Mannitol, 0.5 g/L Dipotassium phosphate, 0.2 g/L Magnesium sulfate, 0.1 g/L Sodium chloride, 1 g/L Calcium carbonate, 0.1 g/L Tryptophan). Following the protocol of Dawwam et al. (2013), 250 µL of cell suspension was inoculated into 5 mL of YEM and incubated at 30°C. After 7 days, 1 mL of cell culture was transferred into a sterile Eppendorf tube and centrifuged at 10,000 rpm for 10 min; then, 500 µL of supernatant was mixed with 1 mL of Salkowski’s reagent and one drop of orthophosphoric acid (85%), and incubated at room temperature for 15 min. A pink coloration indicated the production of indole.

For nitrification, plates of Winogradsky medium (Chatterjee et al., 2015) were inoculated with fresh colonies and incubated at 30°C. After 48h, a drop of Griess’ reagent was added: the test was positive if, after a few seconds, the colonies changed color to pink/purple.

The salt tolerance of all bacterial isolates was estimated based on their ability to grow at different concentrations of NaCl ranging from 5 to 17.5% w/v in NA, incubated at 30°C. NA without salt addition was used as a control. For each microbial isolate, a plating was made on the agarized medium, and the plates were incubated at 30°C for 48h. After incubation, colony development was checked to indicate the ability to grow at the appropriate salt concentration.

Following the protocol suggested by Rashid et al. (2022), for the drought tolerance test, the medium TSB (Trypticase Soya Broth, Oxoid) was modified by adding 5, 10, 15, 20, 25, 30, and 35% of PEG6000 (polyethylene glycol). TSB without the addition of PEG6000 was used as a control. A 1% inoculum (500 µL) was added to test tubes, incubated at 28°C for 48 hours under shaking at 120 rpm. After incubation, the absorbance was read at 600 nm with a UV-VIS spectrophotometer (Hach Lange, Milan, Italy). A medium with different percentages of PEG6000 without the inoculation of microorganisms was used as a control.

### 2.6 Identification of isolates

The most representative isolates were first differentiated at the strain level. For that, total genomic DNA from the samples was extracted and subjected to a Random Amplification Polymorphic DNA- Polymerase Chain Reaction (RAPD-PCR), and the obtained genetic profiles were monitored on 1.5% agarose gels. For the DNA extraction, a single colony was resuspended in 10 µL of MiliQ water. Then, 1 µL of the previously cell suspension was added to 18.7 µL of Master Mix (2 µL 10X Taq buffer; 1.2 µL 50 mM MgCl_2_; 0.4 µL 10 mM dNTPs; 2.5 µL 10 µM M13 primer; 12.6 µL MiliQ water) and subjected to thermal shock (94°C for 4 min). The primer M13 (5’GAGGGTGGCGGTTCT 3’) (Condalab, Barcelona, Spain) was used, given its restrictive action. Subsequently, 0.3 µL of Taq DNA polymerase (Biotools, Madrid, Spain) was added and program run in the thermocycler (2720 Thermal cycler, Applied Biosystems, USA) was performed: 2 cycles with denaturation at 94°C for 1 min, hybridization at 45°C for 1 min and extension at 72°C for 1 min, followed by 35 cycles of denaturation at 94°C for 40 s, hybridization at 52°C for 1 min and extension at 72°C for 3 min, and finally, an extension cycle at 70°C for 4 min. After the reaction, the products were separated by electrophoresis (120 V/1.30 h) on a 1.5% agarose gel and were visualized using RedSafe 20.000X (iNtRON Biotechnology, South Korea) in a gel documentation system by transillumination at 365 nm (Azure biosystem).

For performing the hierarchical dendrograms with the aim of grouping the different strain profiles, the bioinformatics program GelJ v.2 (Heras et al., 2015) was used. The obtained dendrograms (UPGMA, Unweighted Pair Group Method using Arithmetic Average) were based on a similarity matrix (Pearson Similarity Index) generated from RAPD-PCR patterns. Finally, the three most promising candidates were identified to the level of species by amplifying the entire 16S rRNA region with 16S-rRNA Forward primer (5’AGAGTTTGATCCTGGCTCAG3’) and 16S-rRNA Reverse primer (5’AAGGAGGTGATCCAGCCGCA3’) provided by Condalab (Barcelona, Spain). For the amplification, 10 µL of a single colony previously resuspended in MiliQ water was added to 39 µL of Master Mix (18.5 µL MiliQ water, 5 µL 10X Taq buffer; 2 µL 50 mM Mg^2+^; 1 µL 10 mM dNTPs; 6.25 µL 10 µM 16S-rRNA Forward; 6.25 µL 10 µM 16S-rRNA Reverse) and a thermal shock was performed (94°C for 4 min). Subsequently, 1 µL of Taq DNA polymerase (Biotools, Madrid, Spain) was added, and the PCR was performed with the following conditions: 34 cycles with denaturation at 94°C for 1 min, hybridization at 53°C for 1 min, and extension at 72°C for 1 min, followed by final extension at 72°C for 10 min. After the reaction, the products were separated by electrophoresis (100 V/1 h) on 1% agarose gel and were visualized using RedSafe 20.000X in a gel documentation system that made visible the amplified products by transillumination at 365 nm (Azure biosystem). The 16S region from the amplified products was sequenced using the same primers described before (Macrogen Spain, Madrid, Spain). The identities of the obtained sequences were searched in the BLAST database (GenBank, Bethesda, MD, USA). An accession number was assigned to each uploaded sequence to the GenBank database (PX363210, PX363286, PX363300).

### 2.7 Statistical analyses

The analyses were carried out in two independent batches. For each experiment, two technical replications were performed. The results of the qualitative analyses (ammonium production, phosphate-solubilization, silicon solubilization, IAA production, siderophores) were converted into numeric codes (0, negative in all replicates of each isolate; 1, positive in all the replicates) and used as input values to run a principal component analysis; the Euclidean distance was used as the amalgamation method. This statistical analysis was performed using Statistica for Windows (StatSoft, Tulsa, OK, USA).

## 3 Results

**Figure 1:**
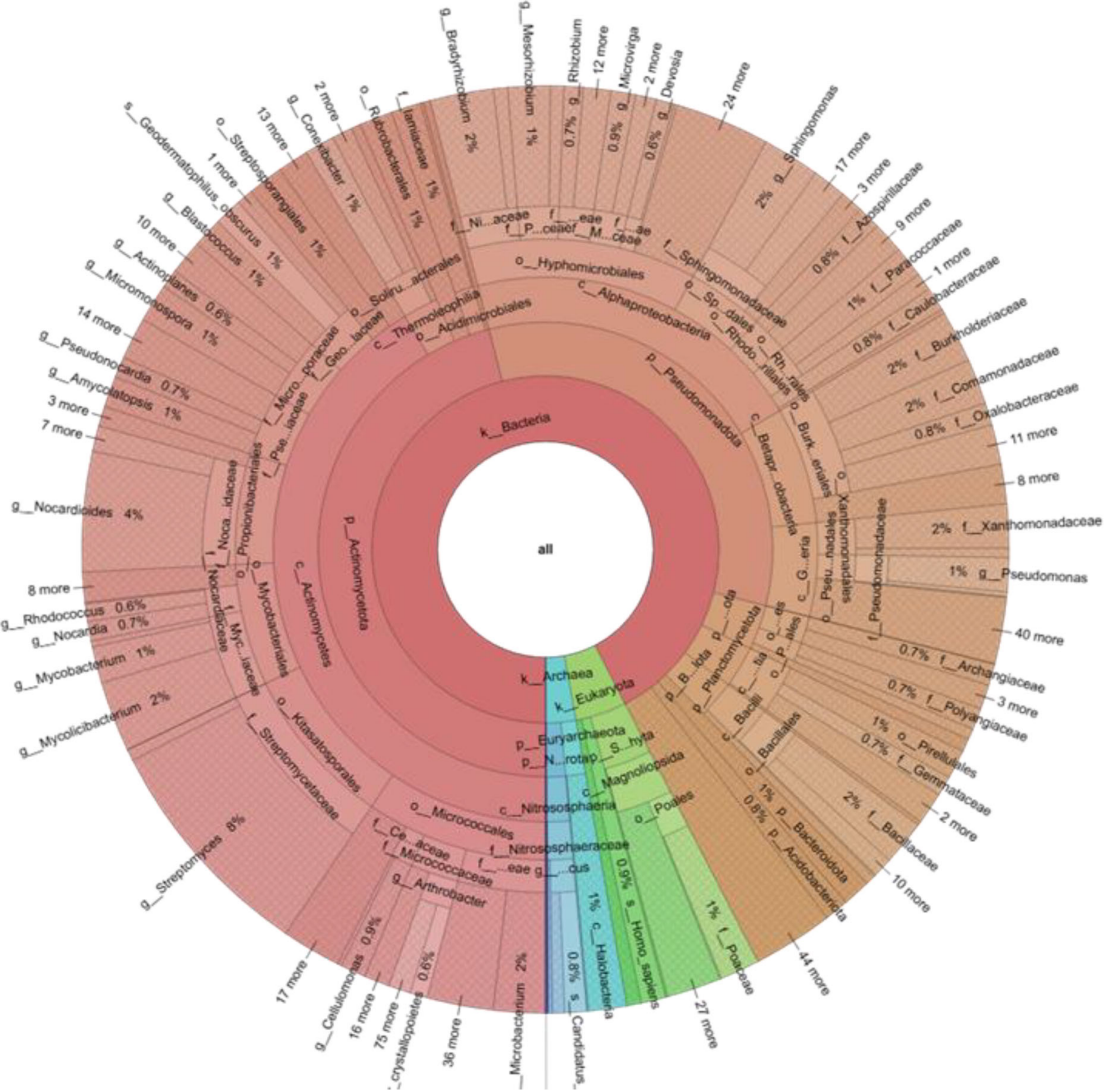
Taxonomic composition of the soil microbial community associated with perennial glasswort (*Arthrocaulon macrostachyum*) rhizosphere. Krona chart showing the relative abundance of microbial domains and their major taxonomic subdivisions based on shotgun metagenomic sequencing of bulk soil samples collected from two sites in Margherita di Savoia (Apulia, Italy) during spring 2023. The chart illustrates the hierarchical taxonomic structure from the domain level (innermost ring) to increasingly specific taxonomic ranks (outer rings).

The metagenomic approach applied to the soil sample allowed us to delineate the compositional structure of the resident microbial community, highlighting a clear predominance of the Bacteria domain (92%), with a minority representation of Eukaryota (5%) and Archaea (3%) (**Figure 1**).

**Figure 2:**
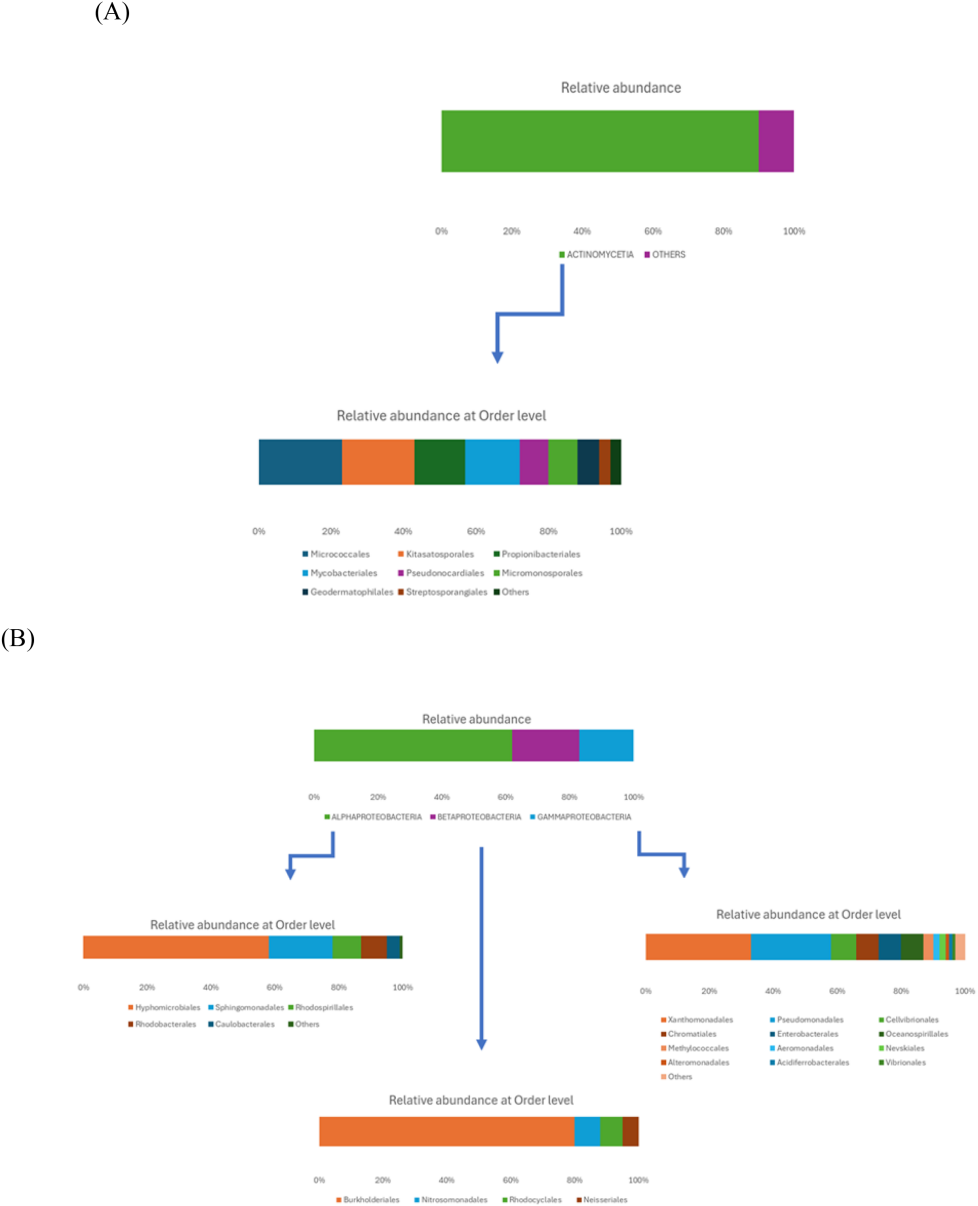
Taxonomic stratification of dominant bacterial phyla in glasswort rhizosphere soil. Stacked bar charts showing the relative abundance (% of phylum reads) of bacterial classes and orders within **(A)** Actinomycetota and **(B)** Pseudomonadota, the two dominant phyla identified through shotgun metagenomic analysis of bulk soil samples from Margherita di Savoia coastal areas (Apulia, Italy).

Phylogenetic analysis of the bacterial component revealed the dominance of two main phyla: Actinomycetota, representing 50% of the bacterial community, and Pseudomonadota, constituting 36% of the population. This taxonomic duality accounted for 86% of the total bacterial diversity (**Figure 1**). Within Actinomycetota, the class Actinomycetia (90%) was dominated by Micrococcales (23%), Kitasatosporales (20%), and Mycobacteriales (15%) (**Figure 2A**). The phylum Pseudomonadota exhibited Alphaproteobacteria (62%) as the majority fraction, dominated by Hyphomicrobiales (58%), while Gammaproteobacteria (17%) showed high taxonomic diversification with Xanthomonadales (33%) and Pseudomonadales (25%) as dominant orders (**Figure 2B**). These taxonomic findings represent the compositional profile of the soil microbiome; functional capabilities were subsequently assessed through culture-dependent phenotypic screening of isolated strains.

To further characterize the cultivable fraction of this diverse microbial community, traditional plate count methods were employed using selective media. Mesophilic and spore-forming bacteria recorded the highest cell density (approximately 7 log CFU/g), while actinobacteria, pseudomonads, and nitrogen-fixing bacteria showed lower counts (approximately 6 log CFU/g). The endophytic population showed a concentration of 3 log CFU/g. From these functional groups, 110 bacterial isolates (100 rhizobacteria and 10 endophytes) were obtained and subjected to systematic phenotypic characterization. Morphological analysis confirmed that while all Pseudomonadaceae isolates were invariably Gram-negative, the remaining bacterial groups (presumed mesophilic, spore-forming, nitrogen-fixing bacteria, and actinobacteria) showed mixed Gram-staining patterns, with approximately 50% displaying Gram-negative morphology. Analysis of the enzymatic response to hydrogen peroxide revealed universal positivity for catalase activity, indicative of aerobic or aerotolerant metabolism. The Pseudomonadaceae family, characterized by a predominantly oxidative metabolism, showed dominant oxidase positivity, while the remaining microbial groups were predominantly oxidase negative. Endophytic bacteria exhibited a Gram-negative, oxidase-negative, and catalase-negative profile.

**Figure 3:**
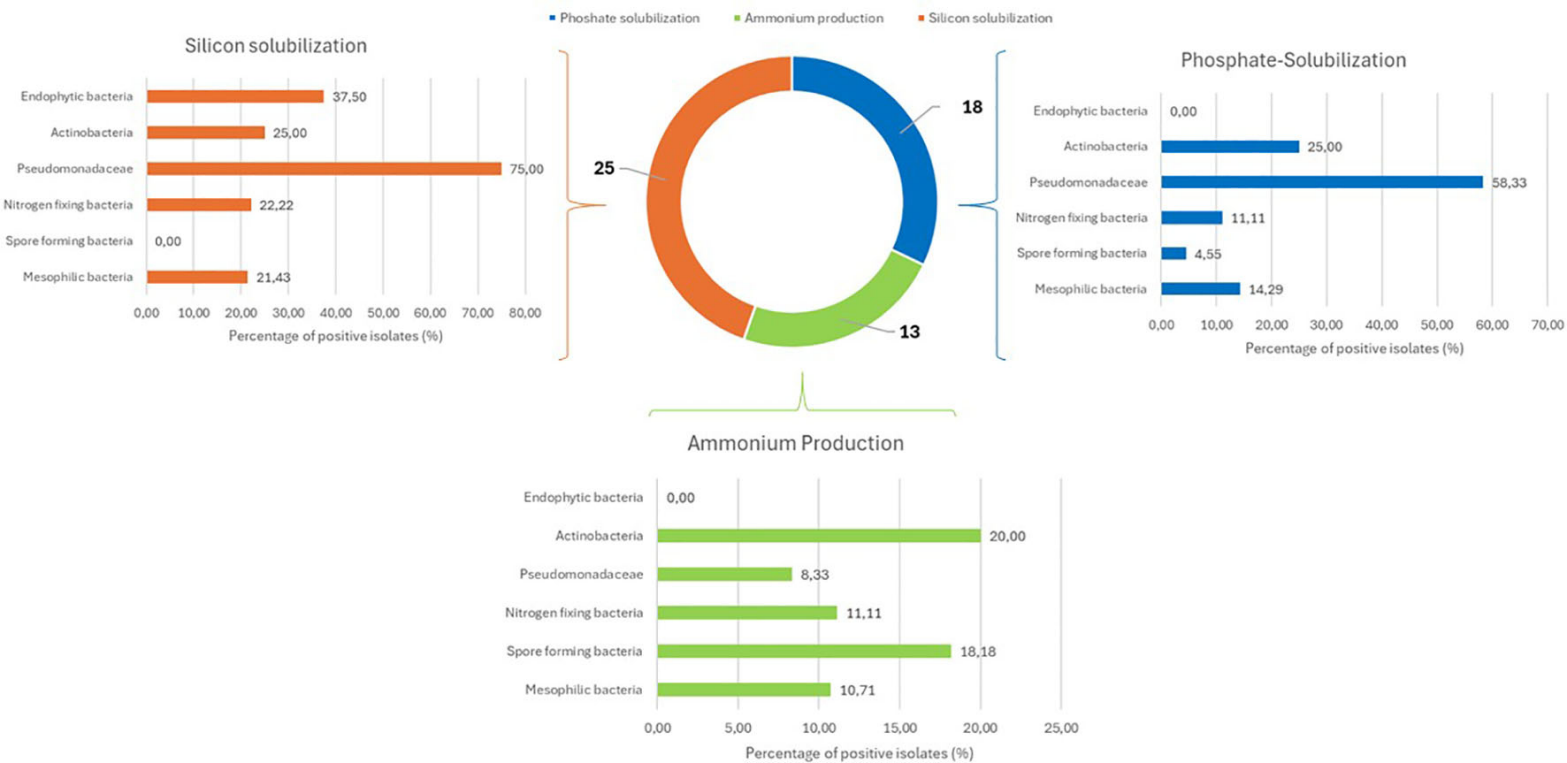
Distribution of plant growth promoting traits among bacterial functional groups isolated from glasswort rhizosphere and endosphere. Bar chart showing the percentage of isolates within each functional group that tested positive for three key PGPB traits: phosphate solubilization (blue bars), silicon solubilization (orange bars), and ammonium production (green bars).Percentages represent the proportion of positive isolates within each functional group.

Functional screening of isolates focused on the characteristic traits of Plant Growth Promoting Bacteria (PGPB). The complete dataset of all PGPB traits for each isolate is provided in **Supplementary Table 1**. Analysis of phosphate and silicon solubilization, along with ammonia production capacity, is summarized in **Figure 3**. Percentages reported below represent the proportion of positive isolates within each functional group. As shown, many isolates exhibited multiple PGPB traits simultaneously, and this multifunctionality is particularly relevant for biotechnological applications. Phosphate solubilization was demonstrated by 18 of the 110 total isolates (16%). Within functional groups, Pseudomonadaceae showed the highest success rate (58%), followed by actinobacteria (25%), mesophiles (14%), nitrogen-fixing bacteria (11%), and spore-forming bacteria (4%). Endophytic bacteria (n=10) were completely devoid of this biochemical capacity.

Silicon solubilization was performed by 25 of 110 strains (23%). Pseudomonadaceae confirmed functional supremacy (75%), followed by endophytic bacteria (38%). Actinobacteria (25%), nitrogen-fixing bacteria (22%), and mesophiles (21%), while spore-forming bacteria (n = 26) were completely ineffective. Ammonia production showed moderate frequencies, with positive responses in 13 of 110 isolates (12%) recording the following results: spore-forming bacteria (18%), mesophiles (11%), Pseudomonadaceae (8%), actinobacteria (20%), and nitrogen-fixing bacteria (11%). Endophytic bacteria (n=10) were completely ineffective.

**Figure 4:**
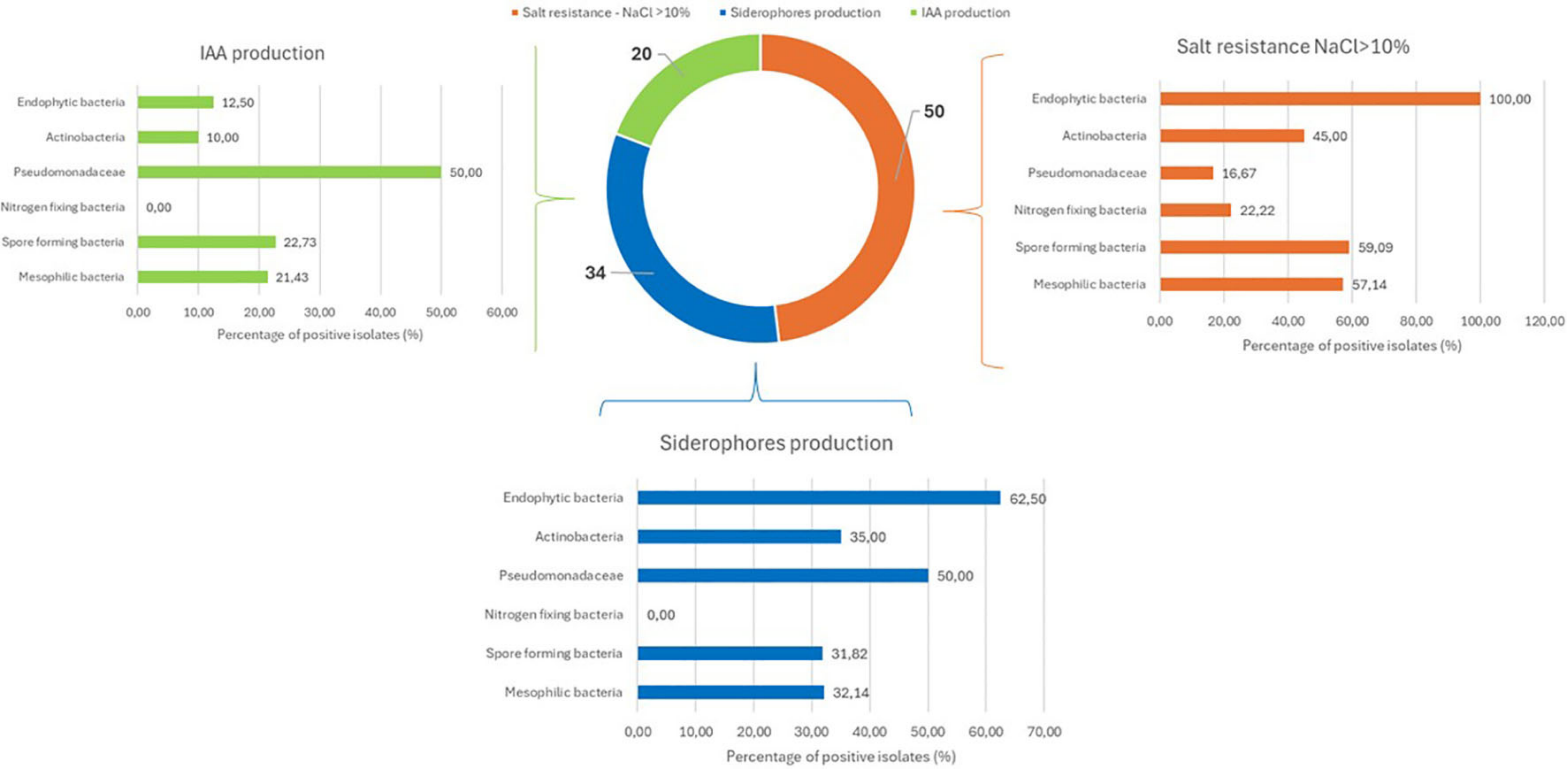
Salt tolerance and additional PGPB traits across bacterial functional groups. Bar chart displaying the percentage of isolates within each functional group exhibiting three traits: salt tolerance (growth at NaCl >10%, orange bars), siderophore production (blue bars), and indole-3-acetic acid (IAA) biosynthesis (green bars). Percentages represent the proportion of positive isolates within each functional group.

**Figure 4** summarizes the results for siderophore and indole-3-acetic acid (IAA) production, combined with salt tolerance data. IAA biosynthesis was detected in only 20 isolates, with the presumptive Pseudomonadaceae predominating (50%). Spore-forming bacteria, mesophiles, endophytes, and actinobacteria showed limited biosynthetic capacity (23%, 21%, 13%, and 10%, respectively), while nitrogen-fixing bacteria were completely inactive. Siderophore production involved 34 isolates, with endophytic bacteria at the top of this trait (63%), followed in decreasing order by Pseudomonadaceae (50%), Actinobacteria (35%), mesophiles (32%), and spore-forming bacteria (31%). Again, nitrogen-fixing bacteria showed complete inactivity.

**Figure 5:**
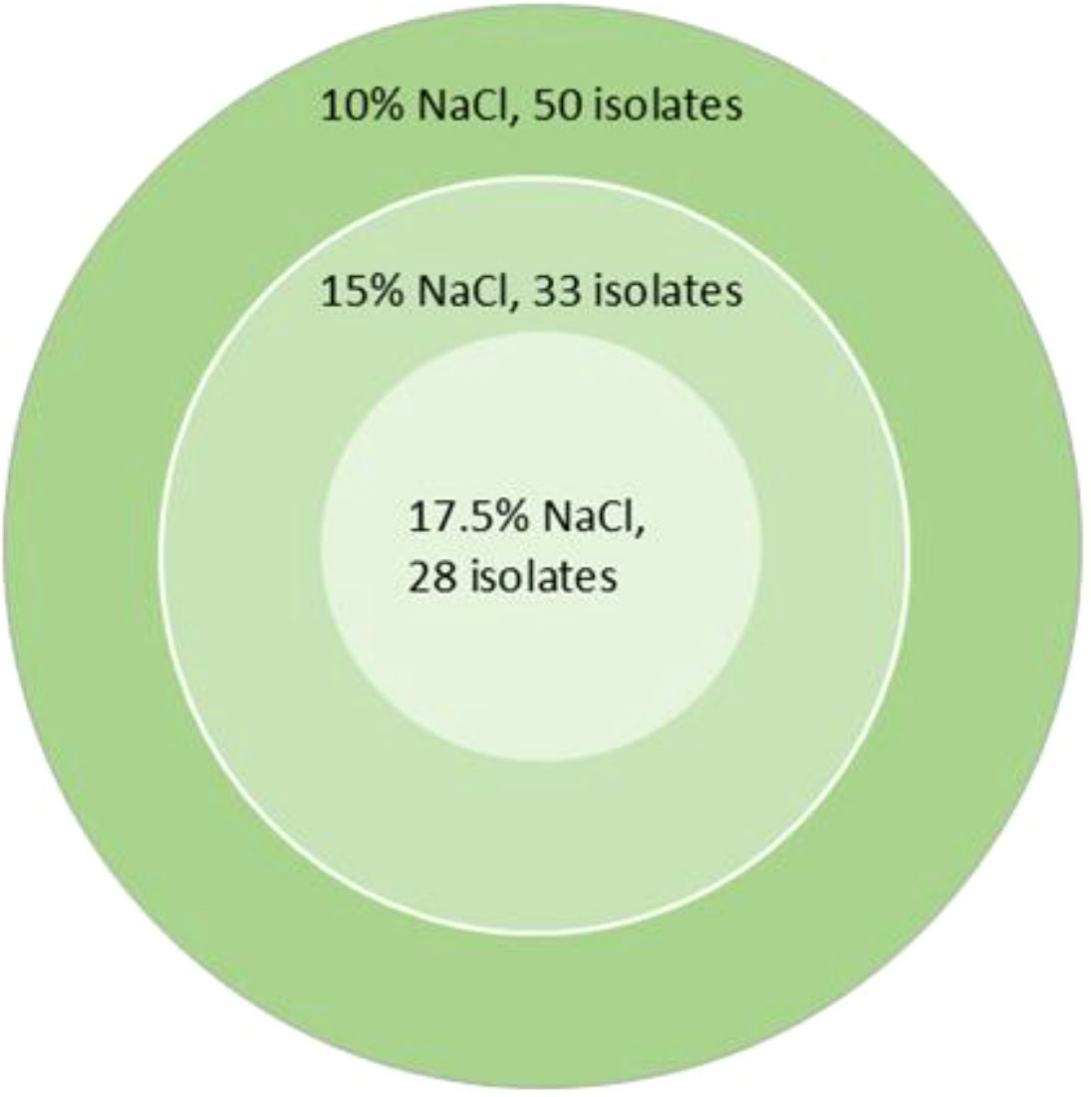
Progressive salt tolerance screening of bacterial isolates at high NaCl concentrations. Venn diagram showing the number of bacterial isolates capable of growth at three increasing NaCl concentrations: >10% (dark green), >15% (medium green), and >17.5% (light green) w/v.

The salt tolerance assay revealed that 50 bacterial strains possess the ability to grow at salt concentrations above 10%. Endophytic bacteria were all salt-tolerant, followed by spore-forming bacteria (59%). Mesophiles and actinobacteria showed remarkable tolerance (57% and 45%, respectively), while nitrogen-fixing bacteria and Pseudomonadaceae showed moderate resilience (22% and 17%). As illustrated in **Figure 5**, 50 strains demonstrated the capacity to grow at salt concentrations exceeding 10%. Among these, 33 strains also showed the ability to thrive at salt concentrations above 15%, while 28 strains displayed exceptional resilience, tolerating salt concentrations as high as 17.5%.

Specific rhizobacterial isolates (4S, 28S, 104S, and 105S) showed exceptional adaptation to water stress conditions, maintaining growth rates up to 50% compared to optimal conditions, making them promising candidates for biotechnological applications in arid environments (data not shown).

**Figure 6:**
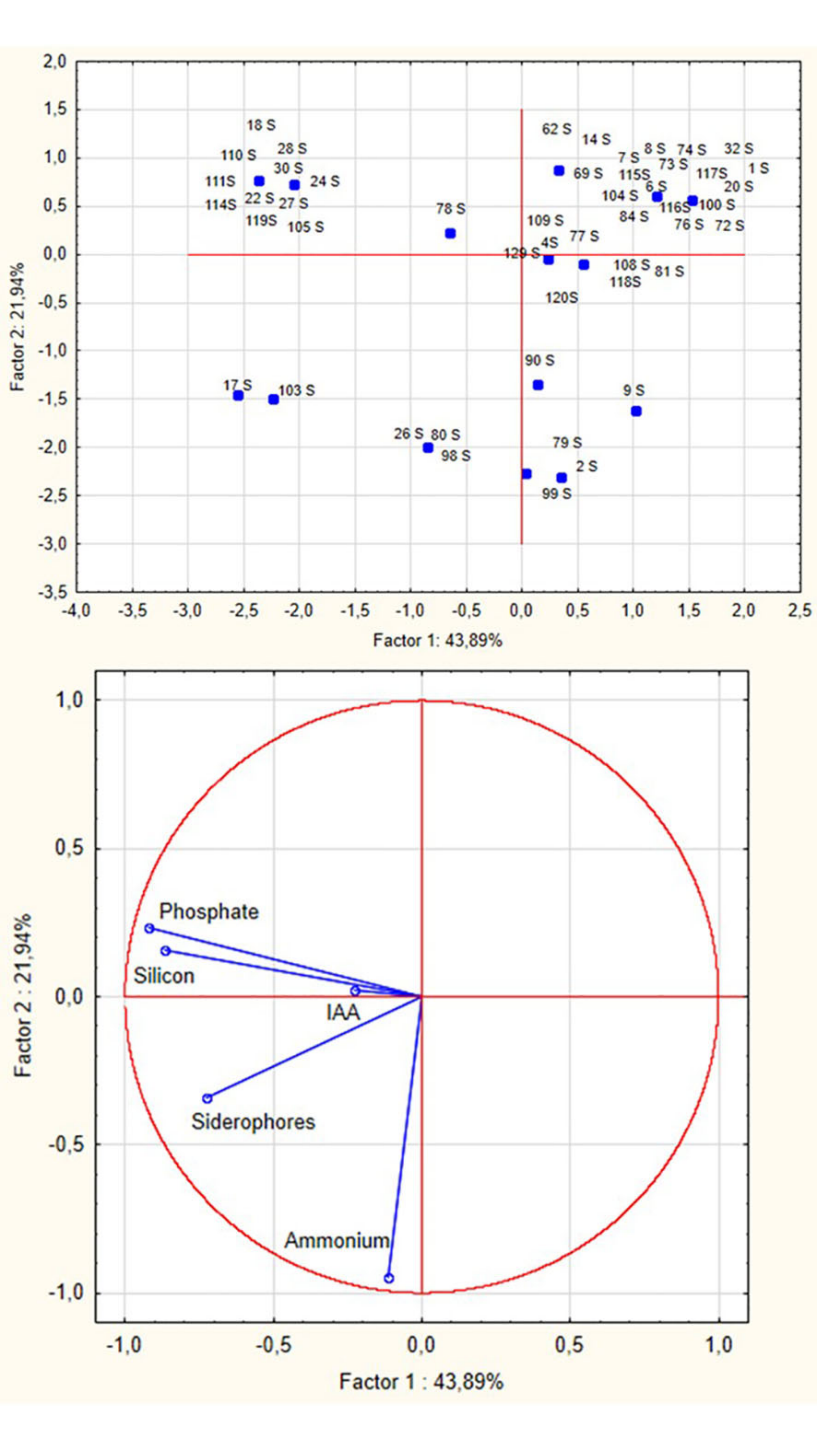
Principal component analysis run on halotolerant bacteria (growth at NaCl>10%). Biplot showing the distribution of 50 halotolerant bacterial isolates (capable of growth at NaCl >10%) along the first two dimensions of Principal Component Analysis. The analysis was performed on binary PGPB trait data (0 = absent, 1 = present), including phosphate solubilization, silicon solubilization, indole-3-acetic acid (IAA) production, siderophore production, and ammonium production. Salt tolerance (>10% NaCl) was used as a selection criterion but not as a variable in the PCA, since all plotted isolates were positive for this trait. Each point represents an individual bacterial isolate, with position determined by its unique combination of PGPB traits. Isolates positioned toward the left side of the plot exhibit multiple positive PGPB traits and represent the most promising candidates for biotechnological applications.

The selection workflow for identifying the most promising PGPB candidates involved multiple sequential steps. First, RAPD-PCR was performed on all isolates to differentiate them at the strain level and identify unique genetic profiles, thereby reducing redundancy in the dataset. Representative strains from each RAPD cluster were then selected for comprehensive PGPB trait characterization. Subsequently, halotolerant isolates (capable of growth at NaCl concentrations >10%) were subjected to Principal Component Analysis (PCA) based on results from qualitative analyses, including assessments of phosphate and silicon solubilization, IAA production, siderophore production, and ammonium production, which were converted into numerical codes and used as input values for principal component analysis (**Figure 6**). PCA identified two principal components explaining 43.89% and 21.94% of the total variance, respectively (cumulative 65.83%). Factor 1 was primarily driven by phosphate and silicon solubilization, siderophore and ammonium production, while Factor 2 was associated with phosphate solubilization and ammonium production. The biplot revealed clear clustering of isolates based on their PGPB profiles, with strains exhibiting multiple positive traits positioned on the left side of the plot.

Among these strains, three isolates were selected based on their comprehensive PGPB profiles (**Table 1**): *Bacillus safensis* (80S) demonstrated positive results for ammonium production, salt tolerance, IAA production, drought tolerance, and siderophore production, but was negative for phosphate and silicon solubilization. *Pseudomonas* sp. (105S) showed positive activity for phosphate solubilization, salt tolerance, silicon solubilization, drought tolerance, and siderophore production, while being negative for ammonium production and IAA synthesis. *Peribacillus frigotolerans* (114S) exhibited the broadest range of PGPB activities, testing positive for all traits except ammonium production.

**Table 1:** Identification and PGPB (Plant Growth Promoting Bacteria) characteristics of the isolates selected.

Isolate	Identification	Accession number	Ammonium production	Phosphate solubilization	Salt tolerance (>10%)	Silicon solubilization	IAA production	Drought tolerance	Siderophores production
80S	*Bacillus safensis*	PX363286	+	–	+	–	+	+	+
105S	*Pseudomonas* sp.	PX363210	–	+	+	+	–	+	+
114S	*Peribacillus frigotolerans*	PX363300	-	+	+	+	+	+	+

**Figure 7:**
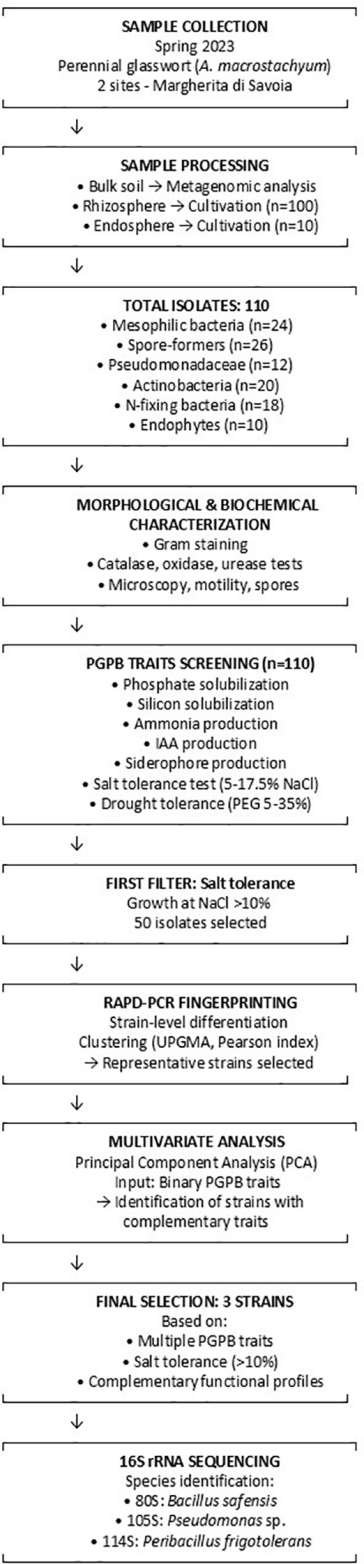
Flowchart of the selection pipeline for identifying promising halotolerant PGPB strains. Starting from 110 bacterial isolates obtained from glasswort rhizosphere and endosphere, strains were sequentially filtered based on salt tolerance (>10% NaCl), subjected to RAPD-PCR fingerprinting for strain differentiation, and analyzed using Principal Component Analysis (PCA) based on PGPB trait profiles. Three final candidates with complementary functional characteristics were selected and identified by 16S rRNA sequencing.

The flowchart of the selection pipeline for identifying the most promising halotolerant PGPB strains is described in **Figure 7**.

## 4 Discussion

The ecological importance of the rhizosphere lies in its function as a nutrient exchange zone between plants and the environment (Yin et al., 2021). Zhou et al. (2024) emphasize that bacteria, constituting generally 94% of the microbial community (a finding also confirmed in the present study), represent key elements for maintaining the functional balance of this complex ecological system. In this study, analysis of the microbial structure revealed a high compositional heterogeneity. The dominant presence of Actinomycetota (with characteristic orders such as Micrococcales, Kitasatosporales, and Streptosporangiales) together with Pseudomonadota, is consistent with microbial communities reported in other saline environments. Members of these taxonomic groups have been previously associated with diverse metabolic capacities, including nitrogen fixation and mineral solubilization (Ma et al., 2021), although the specific functional contributions of these taxa in our samples would require targeted metagenomic or metatranscriptomic analysis (Ma et al., 2021).

The phylogenetic abundance of Actinobacteria and Gammaproteobacteria aligns with previous findings. Marasco et al. (2016) isolated similar PGPB from *Salicornia* rhizosphere in Tunisian coastal areas. The two selected rhizobacteria, also belonging to the *Pseudomonas* and *Bacillus* genera, demonstrated remarkable capabilities: resistance to extreme abiotic stresses, plant hormone synthesis, and stable root colonization. Similar results have also been documented by other researchers (Sadeghi et al., 2012; Bodenhausen et al., 2013; Mukhtar et al., 2017; Etesami and Beattie, 2018; Yamamoto et al., 2018; Kearl et al., 2019; Yin et al., 2021), who identified isolates effective in promoting plant growth under saline conditions.

The taxonomic diversity recorded in the dominant phyla, combined with the metabolic specialization of the orders present, indicated the existence of a structured microbial community with PGPB potential, thus justifying the isolation and characterization of 110 bacterial strains.

The composition of the isolates, characterized by the prevalence of spore-forming Gram-positive bacteria and Gram-negative Pseudomonadaceae, is consistent with patterns commonly observed inin saline environments, where these groups thrive thanks to their sophisticated stress-tolerance mechanisms (Etesami and Beattie, 2018).

A particularly relevant aspect emerges from the analysis of phosphate solubilization, documented in 18 out of 110 isolates. The absence of this ability in endophytes contrasts with what was observed by Santoyo et al. (2021), who suggested a crucial role for these internal microorganisms in phosphate nutrition during salt stress, finding a superior efficacy of endophytes compared to rhizospheric bacteria.

The ability to solubilize silicon, demonstrated by 23% of the isolates (25 out of 110), represents a significant achievement given the role of silicon in strengthening plant defenses against abiotic stress, especially salinity, through cell wall consolidation and optimized water use (Zhu and Gong, 2014).

Regarding IAA production, the limited number of positive isolates (20 out of 110, or 18%) reflects the specialized nature of this function. However, this trait remains strategically important for root development and stress adaptation, conferring biotechnological value to these isolates (Spaepen et al., 2007).

The most notable finding of the study was the high salt tolerance demonstrated by 45% of the isolates (50 out of 110), capable of growth at salt concentrations exceeding 10%. Particularly impressive was the performance of endophytes (all positive), which can be attributed to their adaptation to the internal plant environment, where they must cope with concentrated salt solutions accumulated in plant tissues and maintain osmotic balance under extreme saline stress. The extreme tolerance of 28 strains up to 17.5% salinity suggests the presence of highly specialized halotolerant mechanisms that warrant further investigation and may be potentially exploitable in extreme environmental conditions.

The results obtained confirm the potential of glasswort plants as a reservoir of multifunctional halotolerant PGPB. However, the selection of promising PGPB strains is intrinsically complex, as it requires balancing multiple beneficial traits with strain-specific limitations. Since no single isolate combines all the optimal characteristics, a trade-off–based approach was adopted to identify the most suitable candidates for specific applications. Multivariate statistical analysis, particularly principal component analysis (PCA), was applied to reduce data dimensionality while retaining essential biological information, allowing a more rational selection of strains with complementary functions.

Principal component analysis (PCA) was used to reduce data complexity and identify key variables among PGPB traits (salt tolerance, phosphate and silicon solubilization, IAA, siderophore, and ammonium production) to select the most promising strains. This approach allowed the identification of distinct bacterial groups and the selection of three excellent strains (80S, 105S, 114S) with complementary capabilities.

Their membership in the *Pseudomonas*, *Bacillus*, and *Peribacillus* genera lends particular significance to this selection, considering the recognized PGPB competence and environmental adaptability of these taxa (Gouda et al., 2018; Santoyo et al., 2021). *Pseudomonas* species are distinguished by their versatility in PGPB functions, due to their exceptional metabolic flexibility and ability to employ multiple simultaneous mechanisms for plant growth promotion. In fact, it is well demonstrated that their capacity to combine diverse useful traits, such as siderophore production and phosphate solubilization (also found in this study), makes *Pseudomonas* strains exceptionally valuable in sustainable agriculture, as they can simultaneously enhance plant nutrition, hormone regulation, and disease resistance through multiple complementary pathways (Ganeshan and Manoj Kumar, 2005). On the other hand, *Bacillus* strains offer advantages in terms of environmental persistence and formulation stability, essential characteristics for commercial applications (Walterson and Stavrinides, 2015; Meena et al., 2017). In particular, the selection of *B. safensis* as a PGPB candidate is strongly supported by recent studies demonstrating its effectiveness in promoting plant growth and stress tolerance. Recent research has shown that *B. safensis* can function as a phosphate-solubilizing plant growth promoting bacterium when formulated in carrier-based biofertilizers (Khan et al., 2025), while other studies have documented its ability to promote tomato production under sustainable cultivation conditions and serve as an effective seed coating agent for enhancing plant productivity and stress tolerance (Altimira et al., 2024; Ben-Jabeur et al., 2025). Finally, *Peribacillus frigotolerans* was identified as the third promising strain, with its PGPB characteristics being well-established through extensive scientific evidence. In fact, this species has gained considerable attention in sustainable agriculture due to its ability to produce bioactive compounds and enhance plant stress tolerance via multiple direct and indirect pathways, including IAA production and phosphate solubilization (Świątczak et al., 2024; Romanenko et al., 2024).

Our results align with recent advances in glasswort-associated PGPB. The prevalence of *Pseudomonas* and *Bacillus* confirms findings by Teles et al. (2024) and Sánchez et al. (2025), who reported similar genera with phosphate solubilization and biocontrol capabilities. The multifunctional traits of our selected strains mirror those documented by Cruz de Carvalho et al. (2025) in *S. ramosissima*, reinforcing common patterns in halophyte-microbe associations across different geographical locations. Several lines of evidence support the robustness of our approach. First, the three selected isolates represent genera (*Pseudomonas*, *Bacillus*, *Peribacillus*) with well-established PGPB credentials. Second, recent independent studies have confirmed the effectiveness of the same species we identified as plant growth promoters and stress tolerance enhancers (Khan et al., 2025; Altimira et al., 2024; Ben-Jabeur et al., 2025; Świątczak et al., 2024), providing external validation of our selection outcomes. Third, our deliberate strategy of selecting strains with complementary rather than identical PGPB profiles reflects a pragmatic approach to biotechnological application, recognizing that different mechanisms may be optimal under varying environmental conditions. However, the definitive confirmation of the selection methodology’s success will emerge only from agronomic trials, where the field performance of PCA-selected strains will be systematically compared against non-selected isolates and commercial standards. In fact, despite the comprehensive phenotypic characterization and rational selection of halotolerant PGPB candidates used in this study, several important limitations should be acknowledged. All PGPB traits were assessed under controlled laboratory conditions, and the expression and effectiveness of these traits can vary significantly when bacteria are applied at the plant-soil interface, where complex biotic and abiotic interactions occur (Santoyo et al., 2021). In addition, laboratory-based screening, while essential for identifying promising candidates, cannot fully predict field performance. Finally, our sampling was restricted to two sites within a single geographical area during one season, limiting the generalizability of observed patterns. Therefore, the three selected strains (*B. safensis* 80S, *Pseudomonas* sp. 105S, and *P. frigotolerans* 114S) should be considered as promising candidates requiring validation through plant-based experiments rather than confirmed biofertilizer solutions. The definitive confirmation of our selection methodology’s effectiveness will emerge only from greenhouse and field trials, where the agronomic performance of our PCA-selected strains will be systematically evaluated under salt stress conditions using salt-sensitive crops.

## 5 Conclusions

This study successfully identified and characterized halotolerant bacteria associated with perennial glasswort (*A. macrostachyum*) in Mediterranean coastal ecosystems. Through systematic screening of 110 isolates and multivariate analysis, we selected three promising PGPB candidates (*Bacillus safensis* 80S, *Pseudomonas* sp. 105S, and *Peribacillus frigotolerans* 114S) exhibiting complementary traits including phosphate and silicon solubilization, IAA production, siderophore synthesis, and tolerance to salt (>10% NaCl) and drought stress.

However, this work represents a foundational characterization phase. Laboratory-based trait assessment, while necessary for initial screening, cannot fully predict performance under real plant-soil conditions where complex interactions occur. The ultimate validation of these strains as effective biofertilizers for salt-affected agriculture requires plant-based experiments.

Additional future directions also include: (i) whole-genome sequencing to identify genetic determinants of salt tolerance and PGPB activities; (ii) compatibility studies with native soil microbiomes to evaluate synergistic effects; (iii) expanded sampling (temporal and spatial) coupled with functional metagenomics to capture broader PGPB diversity in coastal halophyte ecosystems. Moreover, comparative microbiome analysis across plant compartments (bulk soil, rhizosphere, rhizoplane, endosphere) using integrated ‘omics approaches (metagenomics, metatranscriptomics, metabolomics) would elucidate compartment-specific taxonomic and functional diversity, differential gene expression patterns across niches, metabolic interactions between rhizospheric and endophytic communities, and tissue-specific contributions to plant salt stress tolerance. Such studies, combined with targeted isolation of underrepresented taxa and plant inoculation experiments comparing strains from different compartments, are strongly suggested to provide mechanistic insights into how microbial spatial organization within the plant holobiont contributes to halophyte salt tolerance strategies.

## Data Availability

The datasets presented in this study can be found in online repositories. The names of the repository/repositories and accession number(s) can be found below: https://www.ncbi.nlm.nih.gov/genbank/, PX363286; https://www.ncbi.nlm.nih.gov/genbank/, PX363210; https://www.ncbi.nlm.nih.gov/genbank/, PX363300.
