# Supplementary material for: Selection and characterization of salt-tolerant plant growth promoting bacteria associated with the endosphere and rhizosphere of perennial glasswort from the Apulia Region (Italy)
Source: Front Plant Sci. 2026 Mar 5;17:1718440. doi: 10.3389/fpls.2026.1718440 (PMC12999581; doi:10.3389/fpls.2026.1718440)
Supplement: Supplementary file 1 [file Table1.docx]

Supplementary Material

**Supplementary Table 1**. PGPB (Plant Growth Promoting Bacteria) characteristics of the 110 isolates selected from perennial glasswort *(Arthrocaulon macrostachyum).* Dark grey shaded cells indicate strains positive for the studied trait.

*Isolate codes are non-consecutive as strains that failed to grow consistently were excluded during preliminary screening.

|  | **Silicon solubilization** | **Phosphate solubilization** | \|  \| \| --- \|   **Ammonium production** | \|  \| \| --- \|   **IAA production** | \|  \| \| --- \|   **Siderophores production** | \|  \| \| --- \|   **Salt tolerance NaCl>10%** |  | **Silicon solubilization** | **Phosphate solubilization** | \|  \| \| --- \|   **Ammonium production** | \|  \| \| --- \|   **IAA production** | \|  \| \| --- \|   **Siderophores production** | \|  \| \| --- \|   **Salt tolerance NaCl>10%** |
| --- | --- | --- | --- | --- | --- | --- | --- | --- | --- | --- | --- | --- | --- | --- | --- | --- | --- | --- | --- | --- | --- |
| \| 1S \| \| --- \| |  |  |  |  |  |  | 56S |  |  |  |  |  |  |
| 2S |  |  |  |  |  |  | 57S |  |  |  |  |  |  |
| 3S |  |  |  |  |  |  | 58S |  |  |  |  |  |  |
| \| 4S \| \| --- \| |  |  |  |  |  |  | 59S |  |  |  |  |  |  |
| 5S |  |  |  |  |  |  | 60S |  |  |  |  |  |  |
| \| 6S \| \| --- \| |  |  |  |  |  |  | 61S |  |  |  |  |  |  |
| \| 7S \| \| --- \| |  |  |  |  |  |  | \| 62S \| \| --- \| |  |  |  |  |  |  |
| \| 8S \| \| --- \| |  |  |  |  |  |  | 63S |  |  |  |  |  |  |
| 9S |  |  |  |  |  |  | 64S |  |  |  |  |  |  |
| 10S |  |  |  |  |  |  | 65S |  |  |  |  |  |  |
| 11S |  |  |  |  |  |  | 66S |  |  |  |  |  |  |
| 12S |  |  |  |  |  |  | 67S |  |  |  |  |  |  |
| 13S |  |  |  |  |  |  | 68S |  |  |  |  |  |  |
| \| 14S \| \| --- \| |  |  |  |  |  |  | \| 69S \| \| --- \| |  |  |  |  |  |  |
| 15S |  |  |  |  |  |  | 70S |  |  |  |  |  |  |
| 16S |  |  |  |  |  |  | 71S |  |  |  |  |  |  |
| 17S |  |  |  |  |  |  | \| 72S \| \| --- \| |  |  |  |  |  |  |
| 18S |  |  |  |  |  |  | \| 73S \| \| --- \| |  |  |  |  |  |  |
| 19S |  |  |  |  |  |  | \| 74S \| \| --- \| |  |  |  |  |  |  |
| \| 20S \| \| --- \| |  |  |  |  |  |  | 75S |  |  |  |  |  |  |
| 21S |  |  |  |  |  |  | \| 76S \| \| --- \| |  |  |  |  |  |  |
| 22S |  |  |  |  |  |  | \| 77S \| \| --- \| |  |  |  |  |  |  |
| 23S |  |  |  |  |  |  | \| 78S \| \| --- \| |  |  |  |  |  |  |
| 24S |  |  |  |  |  |  | \| 79S \| \| --- \| |  |  |  |  |  |  |
| 25S |  |  |  |  |  |  | \| 80S \| \| --- \| |  |  |  |  |  |  |
| 26S |  |  |  |  |  |  | \| 81S \| \| --- \| |  |  |  |  |  |  |
| 27S |  |  |  |  |  |  | \| 82S \| \| --- \| |  |  |  |  |  |  |
| 28S |  |  |  |  |  |  | 83S |  |  |  |  |  |  |
| 29S |  |  |  |  |  |  | \| 84S \| \| --- \| |  |  |  |  |  |  |
| 30S |  |  |  |  |  |  | 85S |  |  |  |  |  |  |
| 31S |  |  |  |  |  |  | 86S |  |  |  |  |  |  |
| \| 32S \| \| --- \| |  |  |  |  |  |  | \| 87S \| \| --- \| |  |  |  |  |  |  |
| 33S |  |  |  |  |  |  | \| 90S \| \| --- \| |  |  |  |  |  |  |
| 34S |  |  |  |  |  |  | \| 100S \| \| --- \| |  |  |  |  |  |  |
| 35S |  |  |  |  |  |  | \| 104S \| \| --- \| |  |  |  |  |  |  |
| 36S |  |  |  |  |  |  | \| 105S \| \| --- \| |  |  |  |  |  |  |
| 37S |  |  |  |  |  |  | \| 108S \| \| --- \| |  |  |  |  |  |  |
| 38S |  |  |  |  |  |  | \| 109S \| \| --- \| |  |  |  |  |  |  |
| 39S |  |  |  |  |  |  | \| 114S \| \| --- \| |  |  |  |  |  |  |
| 40S |  |  |  |  |  |  | \| 115S \| \| --- \| |  |  |  |  |  |  |
| 41S |  |  |  |  |  |  | \| 116S \| \| --- \| |  |  |  |  |  |  |
| 42S |  |  |  |  |  |  | \| 117S \| \| --- \| |  |  |  |  |  |  |
| 43S |  |  |  |  |  |  | \| 118S \| \| --- \| |  |  |  |  |  |  |
| 44S |  |  |  |  |  |  | \| 120S \| \| --- \| |  |  |  |  |  |  |
| 45S |  |  |  |  |  |  | \| 122S \| \| --- \| |  |  |  |  |  |  |
| 46S |  |  |  |  |  |  | \| 124S \| \| --- \| |  |  |  |  |  |  |
| 47S |  |  |  |  |  |  | \| 125S \| \| --- \| |  |  |  |  |  |  |
| 48S |  |  |  |  |  |  | \| 127S \| \| --- \| |  |  |  |  |  |  |
| 49S |  |  |  |  |  |  | \| 129S \| \| --- \| |  |  |  |  |  |  |
| 50S |  |  |  |  |  |  | \| 130S \| \| --- \| |  |  |  |  |  |  |
| 51S |  |  |  |  |  |  | \| 131S \| \| --- \| |  |  |  |  |  |  |
| 52S |  |  |  |  |  |  | 132S |  |  |  |  |  |  |
| 53S |  |  |  |  |  |  | 133S |  |  |  |  |  |  |
| 54S |  |  |  |  |  |  | 134S |  |  |  |  |  |  |
| 55S |  |  |  |  |  |  | 135S |  |  |  |  |  |  |
